# Patterns of sex-specific outcomes and mortality in polytrauma: a demographic and epidemiologic analysis by injury severity score

**DOI:** 10.1007/s00068-025-02930-7

**Published:** 2025-07-07

**Authors:** Vesta Brauckmann, Sophie R. Enke, Anna K. I. M. Dietrich, Claudia Neunaber, Sabine Roth, Michaela Wilhelmi

**Affiliations:** 1 Department of Trauma Surgery, Hannover Medical School, Carl-Neuberg-Straße 1, Hannover, D-30625 Germany; 2 Lower Saxony Center for Biomedical Engineering, Implant Research and Development (NIFE), Stadtfelddamm 35, Hannover, 30625 Germany; 3 Department of Orthopedics, Trauma and Reconstructive Surgery, University Hospital RWTH Aachen, Aachen, Germany

**Keywords:** Polytrauma, Injury severity score, Multiple trauma, Coagulopathy, Critical care, Polytrauma management, Pre-hospital emergency medicine, Trauma care, Trauma center

## Abstract

**Purpose:**

This study evaluates an updated demographic and epidemiological analysis of polytrauma patients, examining sex-specific outcomes, age distribution, and injury severity measured by the Injury Severity Score (ISS).

**Methods:**

This retrospective observational cohort analysis at a level I trauma center in Germany analyzed data from polytrauma patients with an ISS > 16, which were treated in an ICU between 2018 and 2021. Parameters collected included injury scores, pre-hospital data, and clinical outcomes. Assessed was distribution and correlation in pre-hospital and in-hospital outcomes.

**Results:**

In a cohort of 87 polytrauma patients (78.2% male, mean age 45.6 years, mean ISS of 35.4) thoracic injuries were the most frequent (83.9%), followed by injuries of the lower extremity, head, and upper extremity. Females had higher Apache scores and more severe head and neck injuries (*p* < 0.05). Mortality was 15%, deceased patients showing significantly higher ISS. Younger patients had longer hospital stays, averaging 26.1 days. Complications occurred in 90% of patients, predominantly SIRS, followed by kidney failure, ARDS, and sepsis. Prehospital care, including on-scene time, showed no overall correlation with outcomes, except chest drainage, which was associated with higher ARDS and MODS rates. Females received more platelet concentrates, FFPs and TXA.Higher ISS correlated with increased Apache, SOFA, lactate levels and required more blood transfusions and coagulation therapy (*p* < 0.001).

**Conclusion:**

Sex and age were shown to be associated with variations in injury severity, physical response and coagulation management, with females showing distinct injury patterns and physiological burdens. These findings highlight the importance of demographic factors in optimizing polytrauma management and guiding future evidence-based approaches to improve patient care.

**Supplementary information:**

The online version contains supplementary material available at 10.1007/s00068-025-02930-7.

## Introduction

Polytrauma, characterized by multiple severe injuries affecting different organ systems, remains the leading cause of death among individuals under 45 in industrialized countries [1, 2]. In Germany the data from the Trauma Registry of the German Trauma Society (DGU^®^) indicate that 3,815 among 17,283 patients died with Injury Severity Score (ISS) greater than 16, during the acute hospital phase[3] highlighting the critical need for continued research into polytrauma management, epidemiology and outcomes [4].

In this context, research has revealed significant demographic differences in polytrauma outcomes. Studies have shown that older patients experience higher mortality rates and unique injury patterns, driven largely by age-related physiological changes and preexisting conditions [5–7].

Sex differences have been shown to further influence survival outcomes. Studies have demonstrated that females often experience lower rates of mortality, multiple organ failure and infectious complications such as sepsis[8–10] despite similar injury severity compared to male. In women with acute traumatic coagulopathy a twofold higher risk of mortality was found, compared to men [11]. These differences have been partially attributed to biological and hormonal factors [12, 13]. Including estrogen’s anti-inflammatory effects, which may contribute to greater resilience against inflammatory complications, such as systemic inflammatory response syndrome (SIRS) and multiple organ dysfunction syndrome (MODS), which are more frequently observed in male patients [14, 15]. Interestingly, these protective effects persist even after menopause, suggesting that factors beyond hormonal levels contribute to sex-based resilience [15, 16]. Laboratory predictors of trauma mortality, such as base excess, lactate, and coagulation parameters have also been shown to vary by sex, affecting both the immediate response and prognosis after trauma [17].

Body Mass Index (BMI) has emerged as another potentially relevant factor in trauma outcomes, though findings remain inconsistent. While describing an increased risk of infectious complications among patients with higher BMI, it has also been noted that obese patients may paradoxically experience improved survival, despite a higher burden of complications [13, 18, 19].

As for prehospital interventions, such as intubation, fluid resuscitation and hemorrhage control, studies have demonstrated that those early interventions can significantly influence survival rates and long-term outcomes. [4, 9, 20] However, disparities in prehospital interventions, such as underuse of chest tubes in females, have been observed and may contribute to sex-based differences in trauma outcomes [9].

Despite these findings, key gaps remain in understanding how demographic variables, such as age, sex and BMI interact with clinical factors like prehospital management and immune responses to influence outcomes [4, 9]. This study aims provide an updated demographic and epidemiological analysis of severely injured polytrauma patients, with a focus on how sex, age and BMI influence injury severity, prehospital treatment patterns, and early clincal outcomes. In addition, this study examines how preshopital interventions relate to the development of complications. By exploring these variables, the study seeks to contribute to a more nuanced understanding of polytrauma epidemiology and inform future, sex-sensitive strategies to polytrauma care.

## Methods

This retrospective observational, single-center cohort study was conducted at a level I trauma center in Germany from 2018 to 2021, analyzing polytrauma cases treated at the trauma intensive care unit (ICU). This specific time frame was chosen to enable a focused exploratory analysis with complete clinical, laboratory, and prehospital documentation. The study protocol and process of sample donation complied with the Declaration of Helsinki and institutional guidelines. The study has been approved by the Ethics Committee of Hannover Medical School ((Nr. 11650_BO_K_2024).

### Inclusion and exclusion criteria

Patients were selected according to these criteria:

- Inclusion: All patients with ISS > 16 treated at the ICU during the specified period. At our institution all trauma patients with an ISS > 16 are routinely admitted to the ICU.
- Exclusion: Patients younger than 18 were excluded to focus the study on adult polytrauma outcomes.

### Clinical and pre-hospital assessments

This study collected both pre-hospital and in-hospital clinical data, focusing on age and sex-specific differences:

- Demographics and Clinical Variables: Age, biological sex, and body mass index (BMI).
- Injury Severity and Scoring: Injury patterns were classified based on the Abbreviated Injury Scale (AIS) and Injury Severity Score (ISS). The APACHE II score was calculated from initial vitals, lab data, and chronic health conditions to assess the mortality risk, and the SOFA score was recorded on admission to quantify organ dysfunction.
- Pre-Hospital Parameters: The pre-hospital data was analyzed only in the primary admissions-group. Excluded were secondary transfers due to missing data on scene. Response and transport times were recorded to assess any impact on patient outcomes. Administration of medications such as analgesia, sedation, tranexamic acid, catecholamines and others was analyzed. Initial pre-hospital vitals, including heart rate and blood pressure, were collected to calculate the shock index, serving as an early marker of shock and outcome predictor. Pre-hospital invasive procedures such as intubation, chest tube placement, intravenous (IV) catheterization and intraosseous access, as well as other interventions such as immobilizations (e.g., spinal boards), and use of hemostatic agents, were noted.
- Complications and Clinical Outcomes: Complications assessed included SIRS, and sepsis diagnosed following Sepsis-3 criteria, defined by a SOFA score increase of two or more points in cases of confirmed infection.[21]Additional outcomes measured included MODS, acute kidney injury (AKI) according to KDIGO criteria, liver failure as defined by King’s College criteria, and acute respiratory distress syndrome (ARDS) following Berlin criteria [22–24].
- Coagulopathy Particular emphasis was placed on coagulation profiles and management. Coagulation was assessed using standard laboratory values, including prothrombin time (Quick), activated partial thromboplastin time (aPTT), international normalized ratio (INR), fibrinogen levels, and platelet count. Additionally, thromboelastometry was analysed to assess dynamic coagulation status. Coagulopathy management was documented, including tranexamic acid (TXA), blood transfusions, fresh frozen plasma, platelets and the administration of coagulation factors concentrates such as fibrinogen, prothrombin complex concentrate (PCC), antithrombin III (ATIII), and factor XIII. Complications related to coagulation dysfunction, such as disseminated intravascular coagulation (DIC) and bleeding-related mortality were also examined.
- Clinical Measurements: Key outcome measures included ICU length of stay, duration of mechanical ventilation, dialysis, and relevant blood parameters (measured at admission and subsequently at 12, 24, and 48 hours, as well as on days 5 and 14). Included were lactate, base excess, serum-interleukin 6 (S-IL-6), c-reactive protein (CRP), procalcitonin, white blood cells (WBC), haemoglobin, renal markers such as creatinine urea and estimated glomerular filtration rate (eGFR), hepatic function markers such as aspartate aminotransferase (AST) and alanine aminotransferase (ALT). Documented were also catecholamine therapy and volume management.

### Statistical analysis

Data analysis was conducted using SPSS Statistics^®^, Version 29 (IBM SPSS Statistics Corp., New York, NY, USA). Continuous data were presented as mean ± standard deviation (SD), while nominal data were presented as counts and percentages.

Continuous variables (e.g., age, BMI, APACHE II score, SOFA score, laboratory values, ISS) were analyzed using independent samples *t*-tests for group comparisons (e.g., male vs. female, age ≤ 65 vs. >65). Correlation analyses were performed only using the complete dataset for *N* = 80, using cross-tabulations and the Chi-square test for comparisons of dichotomous variables. ISS was analyzed both as a continuous variable and categorically, stratified into ranges (16–30, 31–50, 51–75). While ISS ≥ 16 defines major trauma, thresholds above 30 have been associated with critical outcomes in prior literature[25, 26].

Differences in the ISS between the female and male group were analyzed using t-tests. Association between ISS and outcomes such as complications were analyzed using cross-tabulation and chi-squared test where appropriate. Pearson’s correlation was used to analyze associations between continuous variables such as BMI and renal parameters (eGFR, creatinine), inflammatory markers (e.g., IL-6), and coagulation product administration (e.g., fibrinogen, ATIII). One-way ANOVA was used where comparisons involved more than two groups (e.g., multiple BMI categories).

No multivariable regression analysis was performed due to limited sample size. Instead, potential confounders such as sex and age were explored through stratified analysis and bivariate testing. All statistical tests were two-tailed, with a significance threshold set at *p* < 0.005.

## Results

### Demographic data

A total of 87 patients were analyzed. Of these, 68 (78.2%) were male, and 19 (21.8%) were female. The complete demographical and clinical dataset could only be obtained of 80 patients with 61 male and 19 female. The mean age was 45.7 years (± 20.1), with a range from 18 to 84 years. Age distribution showed a higher proportion of younger patients (18–30 years: 32.5%) and the 51–65 age group (30.0%). Females were slightly older on average (48.21 ± 24.61) compared to males (44.92 ± 18.65). The mean BMI for the cohort was 26.5 (± 4.85), with males having a significantly higher BMI in comparison to females (27.16 ± 4.9 vs. 24.49 ± 4.18, *p* = 0.026). Mortality across the cohort was 13.75% (11 patients), with no significant difference between the female and the male group. Deceased patients had significantly higher ISS scores (*p* = 0.004). (See Table 1) Due to the small number of cases in certain subgroups, such as BMI, age, or AIS-subgroups, statistical testing was not feasible. Therefore, only descriptive values are reported for subgroup comparisons.

**Table 1:** Demographic distribution and statistical comparisons between male and female patients. *N* = 80. BMI = Body mass index. BMI and age in mean ± sd

Item	Total *N* = 80	Male *n* = 61	Female *n* = 19	T-Test/Chi-squared-Test *p*-values
Deceased	*n* = 11 (13,75%)	*n* = 7(11.5%)	*n* = 4 (21.1%)	0.281
BMI (in kg/m ^2^)	26.5 ± 4.85 (18–49)	27.16 ± 4.9 (18–49)	24.49 ± 4.18 (18–35)	0.026
< 18.5	2 (2.5%)	1 (1.6%)	1 (5.3%)	
18.5–24.9	31 (38.8%)	22 (36.1%)	9 (47.4%)	
25–29.9	35 (43.8%)	28 (45.9%)	7 (36.8%)	
30–34.9	8 (10%)	6 (9.8%)	2 (10.5%)	
35–39.9	2 (2.5%)	2 (3.3%)	0 (0.0%)	
> 40	2 (2.5%)	2 (3.3%)	0 (0.0%)	
Age (in years)	45.7 ± 20.1 (18–84)	44.92 ± 18.65	48.21 ± 24.61	0.596
18–30	26 (32.5%)	20 (32.8%)	6 (31.6%)	
30–50	16 (20.0%)	12 (19.7%)	4 (21.1%)	
51–65	24 (30.0%)	21 (34.4%)	3 (15.8%)	
66–80	10 (12.5%)	6 (9.8%)	4 (21.1%)	
81–90	4 (5.0%)	2 (3.3%)	2 (10.5%	

### Injury severity score (ISS) and abbreviated injury scale (AIS)

**Fig. 1:**
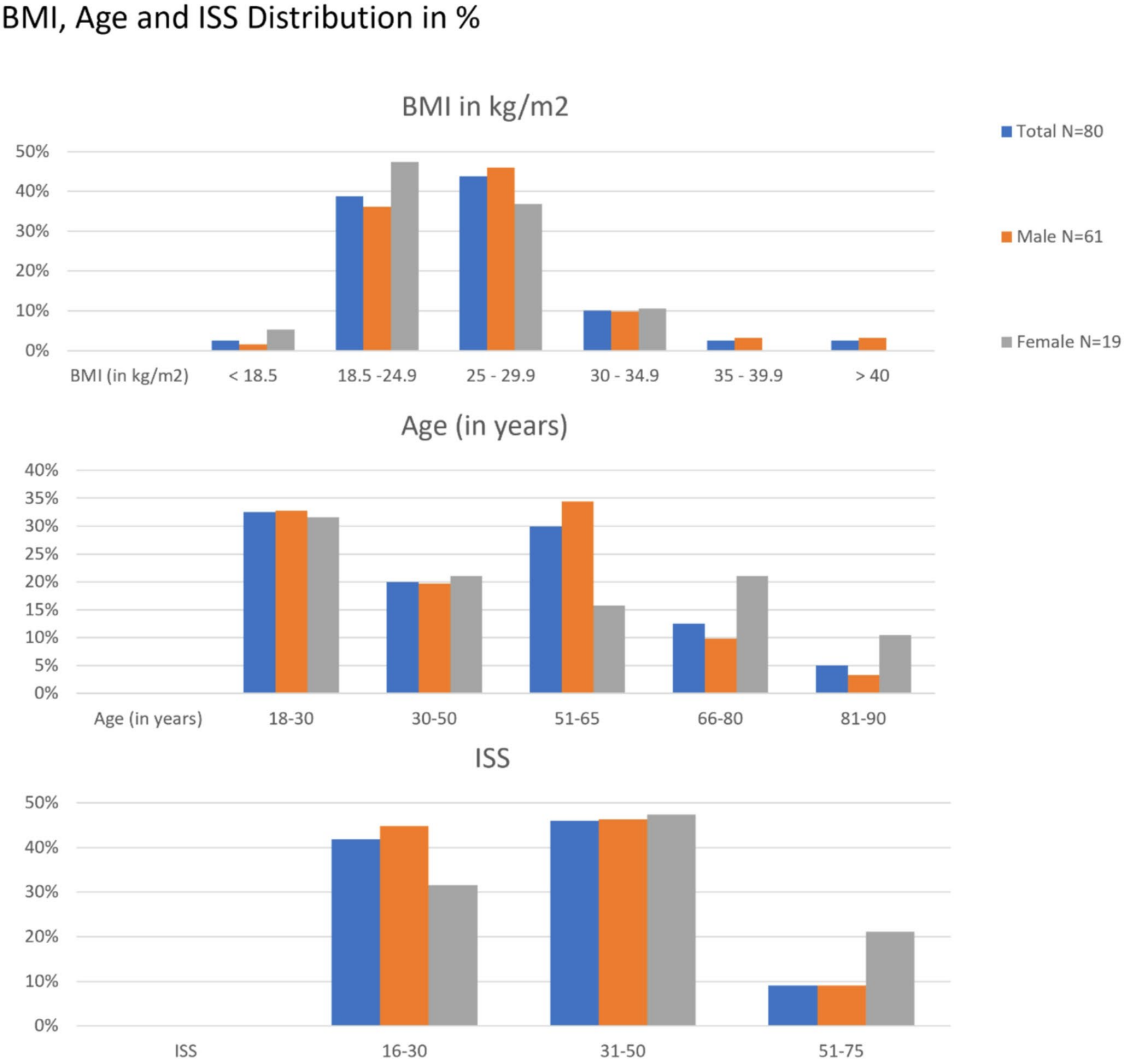
Distribution of Body Mass Index (BMI), Age and Injury Severity Score (ISS) in % by sex

**Fig. 2:**
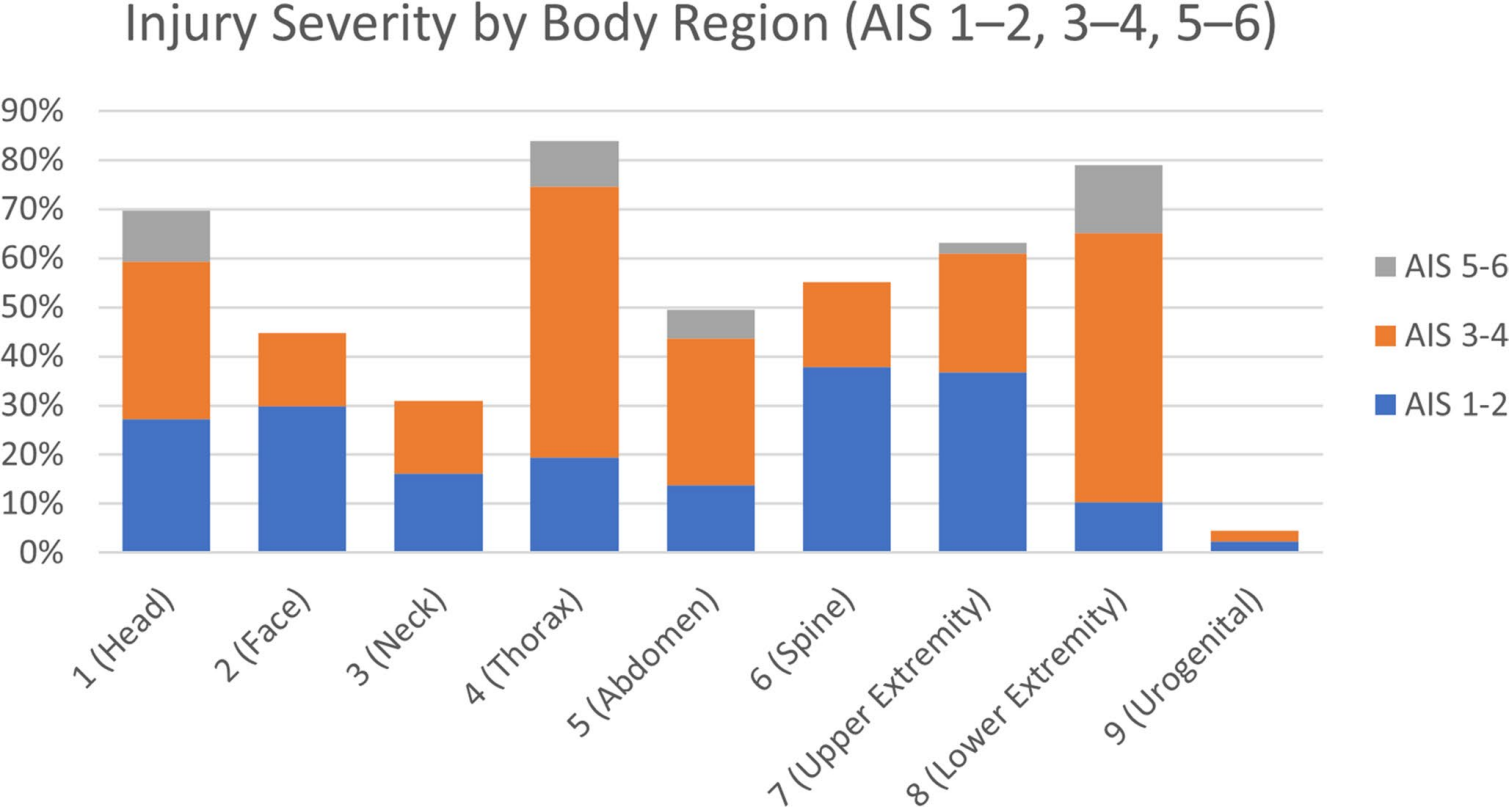
Distribution of Abbreviated Injury Score (AIS) severities by body region. Stacked bars represent the percentage of patients with AIS 1–2, 3–4 and 5–6 injury severity per region

The mean ISS across the cohort was 35.4 (± 13.4). Patients with ISS > 30 comprised 55% of the sample, with higher severity among females (41.37 ± 15.31) compared to males (33.76 ± 12.44, *p* = 0.058). The AIS scores indicated significant injury distribution:

Thoracic injuries were most prevalent with 83.9% with a mean AIS score 2.63 (± 1.48). Second were injuries of the lower extremities including the pelvis with 78.2%, followed by head injuries with 67.8%. Head injuries had a mean AIS score of 1.99 (± 1.81) with females having higher head injury scores compared to males (3 ± 1.9 vs. 1.7 ± 1.7, *p* = 0.013). Then injuries of the upper extremity with 63.2% and spine with 55.2%followed. The AIS groups abdomen with 49.4%, face with44.8%, neck with 31.0% and urogenital with 4.6% were all represented under 50%. Neck injuries averaging an AIS score of 0.55 (± 1.09), being significantly higher in females (1.2 ± 1.51 vs. 0.36 ± 0.85, *p* = 0.028).(See Table 2 and Figs. 1 and 2).

**Table 2:** Distribution of ISS (Injury Severiy score) and AIS (Abbreviated injury Scale) codes and severity of the score

	*n* (%) 10% = *N* 86 Mean ISS/AIS ± SD	Male 100% = *n* = 67 Mean ISS/AIS ± SD	Female 100% = *n* = 19 Mean ISS/AIS ± SD	T-Test
ISS*	35.4 ± 13.4	33.76 ± 12.44	41.37 ± 15.31	0.058
16–30	36 (41.8%)	30 (44.8%)	6 (31.6%)	
31–50	40 (46.0%)	31 (46.3%)	9 (47.4%)	
51–75	10 (9.0%)	6 (9.0%)	4 (21.1%)	
1 (Head)*	59 (67.8%); 1.99 ± 1.81	44 (65.7%) 1.7 ± 1.7	15 (78.9%) 3 ± 1.9	0.013
1–2	22 (27.2)	21 (31.3%)	1 (5.3%)	
3–4	28 (32.2%)	18 (26.9%)	10 (52.6%)	
5–6	9 (10.3%)	5 (7.5%)	4 (21.1%)	
2 (Face)	39 (44.8%); 0.99 ± 1.24	32 (47.8%) 1.01 ± 1.25	7 (36.8%) 0.89 ± 1.24	0.713
1–2	26 (29.9%)	22 (32.8%)	4 (21.1%)	
3–4	13 (14.9%)	10 (14.9%)	3 (15.8%)	
5–6	0 (0%)	-	-	
3 (Neck)*	27 (31.0%); Mean ± SD 0.55 ± 1.09	13 (19.4%) Mean ± SD 0.36 ± 0.85	9 (47.4%) Mean ± SD 1.2 ± 1.51	0.028
1–2	14 (16.1%)	10 (14.9%)	4 (21.1%)	
3–4	13 (14.9%)	3 (4.5%)	5 (26.3%)	
5–6	0 (0%)	-	-	
4 (Thorax)	73 (83.9%); 2.63 ± 1.48	56 (83.6%) 2.57 ± 1.45	17 (89.5%) 2.84 ± 1.61	0.507
1–2	17 (19.5%)	12 (17.9%)	5 (26.3%)	
3–4	48 (55.2%)	40 (59.7%)	8 (42.1%)	
5–6	8 (9.2%)	4 (6.0%)	4 (21.1%)	
5 (Abdomen)	43 (49.4%); 1.63 ± 1.8	31 (46.3%) 1.42 ± 1.73	12 (63.2%) 2.37 ± 1.98	0.069
1–2	12 (13.8%)	11 (16.4%)	1 (5.3%)	
3–4	26 (29.9%)	17 (25.4%)	9 (47.4%)	
5–6	5 (5.7%)	3 (4.5%)	2 (10.5%)	
6 (Spine)	48 (55.2%); 1.09 ± 1.15	36 (53.7%) 1.12 ± 1.21	12 (63.2%) 1.0 ± 0.94	0.651
1–2	33 (37.9%)	22 (32.8%)	11 (57.9%)	
3–4	15 (17.2%)	14 (20.9%)	1 (5.3%)	
5–6	0 (0%)	-	-	
7 (Upper Extremity)	55 (63.2%); 1.52 ± 1.36	44 (65.7%) 1.6 ± 1.39	11 (57.9%) 1.26 ± 1.24	0.321
1–2	32 (36.8%)	25 (37.3%)	7 (36.8%)	
3–4	21 (24.1%)	17 (25.4%)	4 (21.1%)	
5–6	2 (2.3%)	2 (3.0%)	-	
8 (Lower Extremity incl. Pelvis)	68 (78.2%); 2.65 ± 1.69	54 (80.6%) 2.73 ± 1.67	14 (73.7%) 2.37 ± 1.77	0.431
1–2	9 (10.3%)	7 (10.4%)	2 (10.5%)	
3–4	47 (54.9%)	37 (55.2%)	10 (52.6%)	
5–6	12 (13.8%)	10 (14.9%)	2 (10.5%)	
9 (Urogenital)	4 (4.6%); 2.5 ± 0.58	4 (6.0%) 2.5 ± 0.58	-	-
1–2	2 (2.3%)	2 (3.0%)	-	
3–4	2 (2.3%)	2 (3.0%)	-	
5–6	0 (0%)	-	-

### Prehospital data

A majority of patients (75.9%) were primary admissions, while 9.2% were transferred secondarily. Rescue helicopters (RH) transported most patients with 56.3%, followed by an ambulance together with an emergency physician vehicle (EPV). The mean total prehospital time, from alarm to hospital handover was 75.1 min (± 21.16) with only minimal variation between sex groups. The mean on-scene time was 57.7 min (± 18.9).

Initial GCS scores averaged 9.91 (± 5.1), with females presenting slightly lower scores (9.1 ± 4.2) compared to males (10.2 ± 5.4). Prehospital administration of analgesia occurred in 82.4% of patients, with fentanyl being the most common medication and analgesic (70.3%), followed by esketamine (25.7%). Nalbuphine was never used. Tranexemic acid was administered in 23.0% of cases, with a significant sex disparity (males: 28.6% vs. females: 5.6%, *p* = 0.043). Sedation or intubation was performed in 70.3% and 66.2% of cases, respectively, with no significant sex differences. Most commonly midazolam or the combination of midazolam and propofol was used for anesthesia. A muscle relaxant was used in 43.2%. Invasive procedures were performed in 56.3% of cases, a chest tube was documented in 14.9%.

The administration of norepinephrine was documented in 20.3% with a positive shock index documented in 24.3% of the cases. Immobilization techniques were employed in 70.3% of patients, with up to four techniques simultaneously, with vacuum mattresses used in 31.1% and cervical immobilization with stiff necks in half of the study population with 54.1%, showing no significant sex disparities. Noticeable was a difference between the application of pelvic slings; in total they were applied in 20.3% of the cases, but their use less frequent among females. (See Table 3)

**Table 3:** Prehospital data including prehospital time, administered medication, invasive procedures and immobilization. GCS = Glasgow coma scale. CPR = Cardiopulmonary resuscitation

	*N* = 74 = 100% *n* (%)	Male *n* = 56 = 100% *n* (%)	Female *n* = 18 = 100% *n* (%)	*p*-value T-Test/Chi-Squared-Test
Total prehospital time in minutes (between alarm and handover)	75.1 ± 21.16 (37–130 min)	76.0 ± 22.97	72.5 ± 15.14	0.587
Time between arrival at scene and handover in minutes	57.7 ± 18.9 (22–120 min)	58.45 ± 20.71	55.92 ± 13.77	0.650
Initial GCS (mean ± SD)	9.91 ± 5.1	10.2 ± 5.35	9.05 ± 4.21	0.347
Positive Shock-Index	18 (24.3%)	16 (34.8%)	2 (13.3%)	0.114
Administration of any medication	66 (89.2%)	51 (91.1%)	15 (*N* = 18) (83. %)	0.358
Analgesia	61 (90.5%)	48 (85.7%)	13 (72.2%)	0.191
Fentanyl	52 (70.3%)	41 (73.2%)	11 (61.1%)	0.465
Morphine	1 (1.4%)	1 (1.8%)	-	-
Esketamine	19 (25.7%)	17 (30.4%)	2 11.1%)	0.104
Sedation/Anesthesia	52 (70.3%)	40 (71.43%)	12 (66.67%)	0.956
Propofol	11 (14.9%)	9 (16.1%)	2 (11.1%)	0.929
Midazolam	28 (37.8%)	21 (37.5%)	7 (38.9%)	0.916
Etomidate	1 (1.4%)	1 (1.8%)	-	-
Combination of Propofol and Midazolam	12 (16.2%)	9 (16.1%)	3 (16.7%)	0.952
Muscle Relaxant	32 (43.24%)	24 (42.9%)	8 (44.4%)	0.550
Rocuronium	18 (24.3%)	12 (21.4%)	6 (33.3%)	0.592
Succinylcholine	13 (17.6%)	11 (19.6%)	2 (11.1%)	0.169
Vecuronium	1 (1.4%)	1 (1.8%)	-	-
Tranexemic Acid	17 (23.0%)	16 (28.6%)	1 (5.6%)	0.043
Catecholamines	25 (33.8%)	21 (37.5%)	4 (22.2%)	0.104
Adrenaline	1 (1.4%)	-	1 (5.6%)	-
Akrinor	9 (12.2%)	7 (12.5%)	2 (11.1%)	0.875
Noradrenaline	15 (20.3%)	14 (25.0%)	1 (5.6%)	0.085
Invasive Procedures	49 (66.2%)	39 (69.6%)	10 (55.6%)	0.272
Intubation	49 (66.2%)	39 (69.6%)	10 (55.6%)	0.272
Chest Tube	13 (17.6%)	11 (19.6%)	2 (11.1%)	0.640
Peripheral Venous Catheter	67 (90.5%)	52 (92.9%)	15 (83.3%)	0.599
Intraosseous Access	1 (1.4%)	1 (1.8%)	-	-
Immobilization	52 (70.3%)	40 (71.4%)	12 (66.7%)	0.405
Vacuum mattress	23 (31.1%)	18 (32.1%)	5 (27.8%)	0.793
Pelvic Sling	15 (20.3%)	13 (23.2%)	2 (11.1%)	0.360
Stiff Neck	40 (54.1%)	28 (50.0%)	12 (66.7%)	0.172
Head Blocks	0 (0.0%)	-	-	-
Air Chamber splint	4 (5.4%)	3 (5.4%)	1 (5.6%)	0.155
SamSplint	7 (9.5%)	6 (10.7%)	1 (5.6%)	0.155
Traction Splint	1 (1.4%)	1 (1.8%)	-	-
< 1 Immobilization Tool	18 (24.3%)	11 (19.6%)	7 (38.9%)	0.510
Other	20 (27.0%)	15 (26.8%)	5 (27.8%)	0.177
Tourniquet	2 (2.7%)	2 (3.6%)	-	-
Hemostatic Dressing	0 (0.0%)	-	-	-
Repositioning	13 (17.6%)	11 (19.6%)	2 (11.1%)	0.364
CPR	2 (2.7%)	-	2 (11.1%)	-
Packed red blood cells	2 (2.7%)	1 (1.8%)	1 (5.6%)	0.416
Amputation	1 (1.4%)	1 (1.8%)	-	-

### Clinical outcomes

The mean hospital length of stay (LOS) was 26.1 days (± 18.82). Younger patients (≤ 65 years) stayed longer than patients over the age of 65 (28.57 ± 19.69 vs. 15.94 ± 9.8, *p* < 0.001). Complications occurred in 90% of patients, with only 16.3% experiencing more than three complications.

SIRS was developed in 87.5% of the patients, while ARDS and sepsis were observed in 15% and 12.5% of patients respectively. Kidney failure occurred in 31.3%, while MODS and liver failure were documented in less than 5%. Females had seemingly higher rates of mentioned complications; however, those findings were non-significant.

**Fig. 3:**
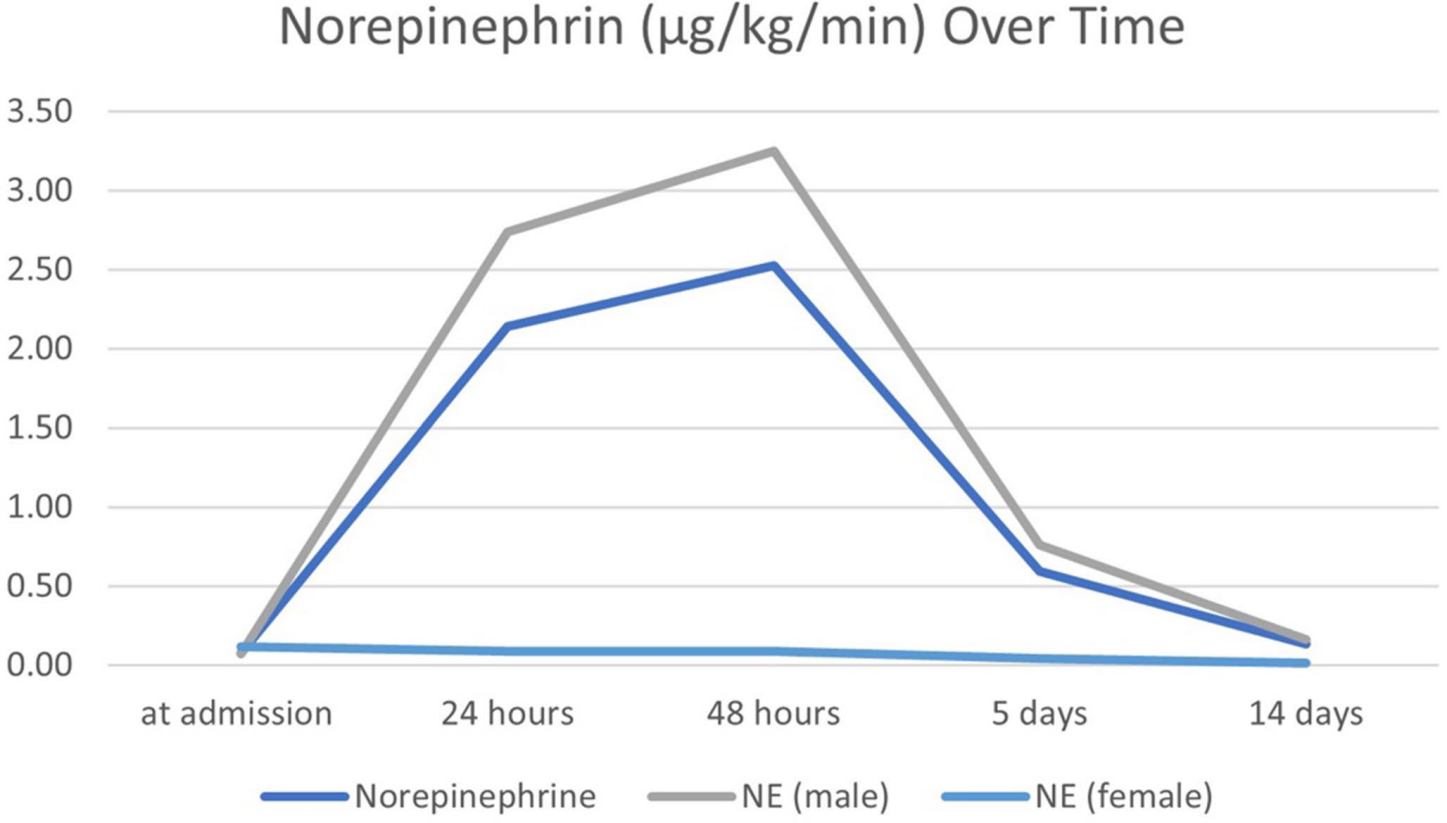
Sex Differences in Norepinephrin (µg/kg/min) Over Time. Time Point at admission, after 24 h, after 48 h, after 5 days, after 14 days

The mean Apache Score was 13.4 (± 6.7) and significantly higher in females (16.53 ± 6.56) than males (12.46 ± 6.5, *p* = 0.03). The SOFA score averaged 8.14 (± 2.8) and showed no significant sex differences. Cardiopulmonary resuscitation (CPR) was performed in five patients (6.3%), with females undergoing CPR slightly more frequently (15.8%) than males (3.3%). Return of spontaneous circulation (ROSC) occurred in three (3.8%) cases, predominantly in females (10.5% vs. 1.6%). Dialysis was required for a mean duration of 1.9 days (± 6.6). The mean duration of intubation was 327.3 h (± 314.8). (See Table 4). Norepinephrine dosage increased until 48 h after admission, females showed lower dosage, however this was not considered statistically significant (See Fig. 3). The number of surgical interventions averaged five per patient (± 5), ranging from 0 to 33.

**Table 4:** Clinical characteristics, outcomes, and statistical comparisons between male and female patients.sirs = systemic inflammatory response syndrome, sofa = sequential organ failure assessment, mods = multiple organ dysfunction syndrome. ARDS = Acute respiratory distress syndrome. CPR = Cardiopulmonary resuscitation. ROSC = Return of spontaneous circulation

Item	Total *N* = 80	Male *n* = 61	Female *n* = 19	T-Test/Chi-squared-Test *p*-values
Apache Score	13.4 ± 6.7	12.46 ± 6.5	16.53 ± 6.56	0.026
Complications	72 (90%)	54 (88.5%)	18 (94.7%)	0.197
< 3	59 (73.8%)	46 (75.4%)	13 (68.4%)	-
3–6	13 (16.3%)	8 (13.1%)	5 (26.3%)	-
SIRS	70 (87.5%)	52 (85.2%)	18 (94.7%)	0.275
SOFA-Score	8.14 ± 2.8	8 ± 3.04	8.58 ± 1.92	0.330
Sepsis	10 (12.5%)	6 (9.8%)	4 (21.1%)	0.197
MODS	4 (5%)	2 (3.3%)	2 (10.5%)	0.206
Kidney Failure	25 (31.3%)	19 (31.1%)	6 (31.6%)	0.972
Liver Failure	1 (1.3%)	1 (1.6%)	0 (0.0%	0.574
ARDS	12 (15%)	10 (16.4%)	2 (10.5%)	0.532
CPR	5 (6.3%)	2 (3.3%)	3 (15.8%)	0.083
ROSC	3 (3.8%)	1 (1.6%)	2 (10.5%)	0.709
Dialysis (in days)	1.9 ± 6.6 (0–45)	1.53 ± 4.99	3.11 ± 10.34	0.529
Duration of Intubation (in hours)	327.3 ± 314.8 (0–1540)	307.45 ± 267.8	390.98 ± 436.35	0.438
Length of hospital stay (in days)	26.1 ± 18.82	27.59 ± 19 − 8	20.79 ± 13–96	0.097

Among the 11 patients who died during hospitalization, the mean age was 46.73 ± 25.8 years, BMI 26.6 ± 8.1, ISS 49.36 ± 18.21, and APACHE II score 19.0 ± 7.0. They experienced on average 2.6 ± 1.9 complications. The average length of stay until death was 5.82 ± 3.7 days (range 2–15); nine patients died within the first week.

Significant associations with mortality were observed for ISS (*p* < 0.001), APACHE II (*p* = 0.003), SOFA (*p* = 0.016), number of complications, MODS, and resuscitation (all *p* < 0.001). No significant associations were found for sex (*p* = 0.281), age, BMI, SIRS, or sepsis.

### Laboratory findings

Laboratory parameters revealed following key trends. IL-6 levels (ng/l) peaked at admission (mean 1385.3 ± 2168.3) and decreased over time, with significant differences between the female and the male group at 14 days. Men had higher values (159.86 ± 159.74) then women (51.67 ± 37.41) (*p* = 0.008). WBC counts (10^3/µl) were elevated in females at 48 h (12.6 ± 3.5) and 5 days (11.0 ± 3.0) compared to males (10.1 ± 3.71 at 48 h and 8.84 ± 2.53 at 5 days) (*p* = 0.01 for both). Admission hemoglobin levels were significantly lower in females (10.3 ± 2.4) than males (11.9 ± 2.1, *p* = 0.015). INR values at 14 days showed differences (female 1.0073 ± 0.065 vs. male 1.0734 ± 0.125, *p* = 0.025). Creatinine levels (µmol/l) varied significantly across sexes until 12 h after admission and after 5 days with no significant difference in eGFR rates. Females had lower creatinine levels (at admission 81.79 ± 27.16 vs. 100.75 ± 28.41, *p* = 0.013; at 12 h 79.94 ± 24.16 vs. 95.33 ± 22.71, *p* = 0.033; at 5 days 72.83 ± 39.62 vs. 103.74 ± 87.06, *p* = 0.045; at 14 days 68.17 ± 45.23 vs. 117.3 ± 118.03, *p* = 0.041). No other laboratory parameter showed significant differences between sexes. The trends over time of all laboratory parameters are visualized in supplementary data 1.−3.

### Coagulopathy, blood product administration, and coagulation therapy

Coagulopathy was identified in a large proportion of patients. Blood product use included red blood cells; mean transfusions were 11.4 (± 10.4) units per patient, similar across sexes. Females received more platelet concentrates (2.95 ± 5.1 vs. 1.5 ± 2.7) and required higher volumes of fresh frozen plasma (4.9 ± 7.6 vs. 3.95 ± 5.3), though not statistically significant.

**Fig. 4:**
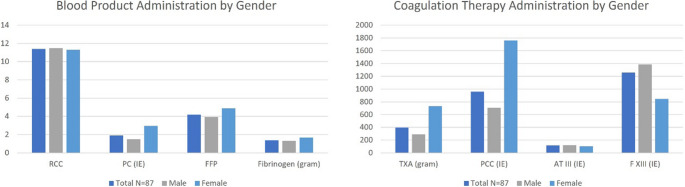
Blood Product and Coagulation Therapy Administration by Sex. Abbreviations: RCC = red blood cell concentrates; PC = platelet concentrate; FFP = Fresh Frozen Plasma; TXA = tranexemic acid; PCC = prothrombin complex concentrate; AT III = Antithrombin III; F XIII = fibrin stabilizing factor XIII. IU = International Units

Fibrinogen was administered in 33.75% of the cases with a mean dosage of 1.4 g (± 2.6). Patients additionally received an average of 957.9 IU (± 2238.67) of Prothrombin Complex Concentrate (PCC), and 396.87 ± 998.93 mg of tranexamic acid, with females receiving more of fibrinogen, PCC and tranexamic acid, this finding was statistically non-significant (*p* = 0.584, *p* = 0.261 and *p* = 0.298, respectively). No significant difference was found in antithrombin and factor XIII administration. (See Table 5; Fig. 4)

**Table 5:** Blood product and coagulation therapy administration (in total) by sex. RCC = Red cell concentrate, pc = platelet concentrate, ffp = fresh frozen plasma, pcc = prothrombin complex concentrate, txa = tranexemic acid, atiii = antithrombin III, F xiii = fibrin stabilizing factor, iu = international units

	*N* = 87	Male	Female	t-Test
Red Cell Concentrate (RCC) (in units)	11.4 ± 10.4	11.5 ± 10.1	11.3 ± 11.8	0.95
Platelet Concentrate (PC) (in units)	1.9 ± 3.4	1.5 ± 2.7	2.95 ± 5.1	0.25
Fresh Frozen Plasma (FFP) (in units)	4.2 ± 5.9	3.95 ± 5.3	4.9 ± 7.6	0.617
Fibrinogen (in gram)	1.4 ± 2.6	1.3 ± 2.4	1.7 ± 3.2	0.584
Prothrombin Complex Concentrate (PCC) (in IU)	957.9 ± 2238.67	707.1 ± 1326.5	1763 ± 3902.9	0.261
Tranexemic Acid (TXA) (in gram)	396.87 ± 998.93	292.6 ± 583.0	731.8 ± 1758.2	0.298
Antithrombin III (ATIII) (in IU)	117.28 ± 434.97	121.0 ± 431.2	105.2 ± 458.8	0.896
Fibrin stabilizing factor (F XIII) (in IU)	1256.61 ± 1816.81	1384.7 ± 1979.5	845.2 ± 1091.5	0.809

### Correlation analysis

Correlation analysis was only performed for patients with a complete dataset (*N* = 80). Higher ISS (> 30) correlated significantly with higher Apache scores (*p* = 0.03), higher SOFA scores (*p* < 0.001), elevated lactate (*p* < 0.001), and lower base excess (*p* = 0.001). It also correlated with higher PCT levels (*p* = 0.017), lower hemoglobin (*p* < 0.001), reduced PTT (*p* < 0.001), and decreased eGFR after 48 h (*p* = 0.004). Patients with higher ISS received significantly more blood transfusions (*p* < 0.001), platelet concentrates (*p* < 0.001), FFPs (*p* < 0.001), fibrinogen (*p* = 0.014), PPSB (*p* = < 0.001), tranexamic acid (*p* = 0.063) and Factor XIII (*p* < 0.001). A positive shock index in the prehospital phase was associated with higher ISS (*p* = 0.003).

Older age (> 65 years) also correlated with higher Apache scores (18 ± 6.4 vs. 12.4 ± 6.4, *p* = 0.006), higher creatinine levels at day 14 (*p* = 0.04), and reduced eGFR after 48 h (*p* < 0.001). Higher BMI was associated with lower eGFR during the first 12 h post-admission (*p* = 0.045).

Analysis of prehospital time showed no significant impact on clinical outcomes. Cross-tabulations indicated no correlation between prehospital medication, a positive shock index, and complications such as SIRS, sepsis, MODS, ARDS, or CPR. However, prehospital intubation and invasive measures correlated with ARDS (Pearson *p* = 0.028), but not with other complications. Prehospital chest tube insertion was associated with MODS (*p* = 0.024) and ARDS (*p* = 0.042), as well as CPR (*p* < 0.001) and increased complications (*p* = 0.037).

## Discussion

This study aimed to evaluate demographic and epidemiological characteristics of polytrauma patients treated at a level I trauma center, with a particular focus on sex, age, and BMI-related differences in clinical outcomes. Additionally, the study explored the role of prehospital management on survival and complication rates.

The demographic profile of our cohort aligns with national and global polytrauma statistics, characterized by a predominance of younger male patients and a mean age of mid-forties, consistent with prior studies [3, 27, 28]. Females were, on average, slightly older than males, aligning with previous findings that older females are more prone to severe injuries due to factors such as reduced bone density [5, 6]. BMI distribution was shown to have significantly higher values among males, consistent with global trauma registries [11, 13]. The mean Injury Severity Score (ISS) of 35.04 in our cohort was notably higher than the ISS range of 25 to 29 as reported in similar studies [28, 29], potentially reflecting differences in injury severity at our center. Thoracic injuries were most prevalent across both sexes, followed by head and lower extremity injuries, consistent with global trends [4, 20]. However, in our study females exhibited significantly higher Abbreviated Injury Scale (AIS) scores for head and neck injuries. This finding contrasts with previous literature where it has been suggested that males are more frequently involved in high-impact mechanisms, such as road traffic accidents or occupational injuries, leading to a greater incidence of traumatic brain injuries [4].

In contrast to previous literature, our data showed no significant impact of prehospital management on overall outcomes. While timely interventions, such as intubation, tranexamic acid (TXA) administration, and hemorrhage control, have been associated with improved outcomes [4, 9, 30], our results suggest that their efficacy may be influenced by injury severity rather than the interventions themselves. For instance, the observed correlation between chest tube placement and acute respiratory distress syndrome (ARDS) or multiple organ dysfunction syndrome (MODS) may be attributed to the high incidence of thoracic trauma in our cohort rather than the procedure itself [20]. Notably, the use of pelvic slings was lower in females despite comparable injury patterns and no difference in the severity of pelvic injuries. This discrepancy may indicate unconscious biases or misperceptions of injury severity during prehospital management [14, 31, 32]. No lower numbers of cervical immobilization measurements in females was seen, which does not align with reports of under-recognition of potential spinal injuries in this group and a less likely aggressive prehospital care [14, 31].

Interestingly, younger patients in our cohort had significantly longer hospital stays compared to older individuals, despite similar ISS scores. While literature often associates prolonged recovery with advanced age due to comorbidities and diminished physiological reserves [5, 6]. One possible explanation might be that younger patients might undergo more aggressive surgical interventions and rehabilitation programs, extending their length of stay but potentially improving long-term outcomes [9].

Sex-specific differences were evident in our study. Higher APACHE scores in females suggest a greater physiological burden at admission, potentially influenced by lower baseline hemoglobin levels and reduced renal function [8, 15]. Hormonal differences, such as estrogen’s modulation of the immune response, may also contribute to the higher IL-6 and white blood cell levels observed in females [33]. Prior literature has also noted that females, particularly older ones, may have higher rates of conditions such as anemia or osteoporosis, which could further compound their physiological burden [34, 35]. Additionally, our findings showed a higher, but not significantly, incidence of sepsis in females, which stands in contrast to the usually described fewer complications and better outcomes in females [8, 9, 15]. One might argue, that while male sex hormones are generally considered immunosuppressive and female sex hormones immunoprotective [36, 37], contrasting studies have noted higher in-hospital sepsis-related mortality in females [38]. The higher prevalence of ARDS in males, albeit not statistically significant, aligns with previous findings [39, 40]. Moreover, contrasting with our findings, better renal function and lower MODS rates in females were reported [9, 10], while our cohort demonstrated reduced renal function and no significant sex differences in complications rates. The differences observed in other studies may be influenced by variations in prehospital care, baseline health conditions, or population-specific factors.

Coagulopathy was prevalent in our cohort, necessitating significant transfusion of blood products and coagulation therapies. The mean transfusion requirement in our cohort was 11.4 units of packed red blood cells (RBCs), exceeding the averages reported in prior studies (3-10.5 units) [11, 41, 42]. Females required more fibrinogen, prothrombin complex concentrate (PCC), and TXA, likely due to their lower baseline hemoglobin levels and higher risk of coagulopathy. These findings diverge from reports of lower TXA administration rates among females [30].

Patients with ISS > 30 exhibited significantly higher APACHE and SOFA scores, elevated lactate levels, and increased transfusion requirements, corroborating their elevated mortality risk. These findings are consistent with studies showing a correlation between higher ISS and worse outcomes, emphasizing the critical role of injury severity in predicting outcomes [4]. Elevated inflammatory markers and reduced base excess further highlight the systemic impact of severe injuries [33, 43]. The New Injury Severity Score (NISS) might offer more precise predictions than ISS [44, 45]. However, ISS remains the standard for trauma severity assessment [46].

This study’s retrospective design and single-center setting limit the generalizability of our findings. The relatively small sample size, particularly for females, may have reduced statistical power. Additionally, no multivariate adjustment was applied to control for potential confounders. Consequently, observed associations—particularly those involving BMI, sex, and age—may reflect interrelated effects. Long-term outcomes and rehabilitation data were not captured. Exclusion of patients who did not survive to hospital admission introduces potential selection bias. Future multicenter studies with larger, more diverse cohorts are essential to validate these findings and elucidate the mechanisms underlying sex, BMI, and age-related differences in polytrauma outcomes. Prospective studies incorporating molecular and immunological analyses could further advance understanding of trauma recovery.

## Conclusion

This study highlights significant demographic, sex, and age-related differences in polytrauma outcomes within a single-center cohort. While younger males were the majority, females exhibited distinct injury patterns and higher physiological burdens. Sex disparities in prehospital care were not significant, however differences in the clinical management were evident, indicating potential biases. Age influenced outcomes, with younger patients having longer hospital stays. Although injury severity strongly predicted outcomes, prehospital interventions had limited impact on survival. Future multicenter studies are crucial to validate these findings and guide personalized, evidence-based approaches to improve polytrauma care.

## Supplementary information

Below is the link to the electronic supplementary material.

ESM 1 (DOCX 496 KB)

## Data Availability

The data that support the findings of this study are available from the corresponding author upon reasonable request.
