# Supplementary material for: Patterns of sex-specific outcomes and mortality in polytrauma: a demographic and epidemiologic analysis by injury severity score
Source: Eur J Trauma Emerg Surg. 2025 Jul 7;51(1):250. doi: 10.1007/s00068-025-02930-7 (PMC12234584; doi:10.1007/s00068-025-02930-7)
Supplement: Supplementary file 1 — (DOCX 496 KB) [file 68_2025_2930_MOESM1_ESM.docx]

## Supplementary Data:


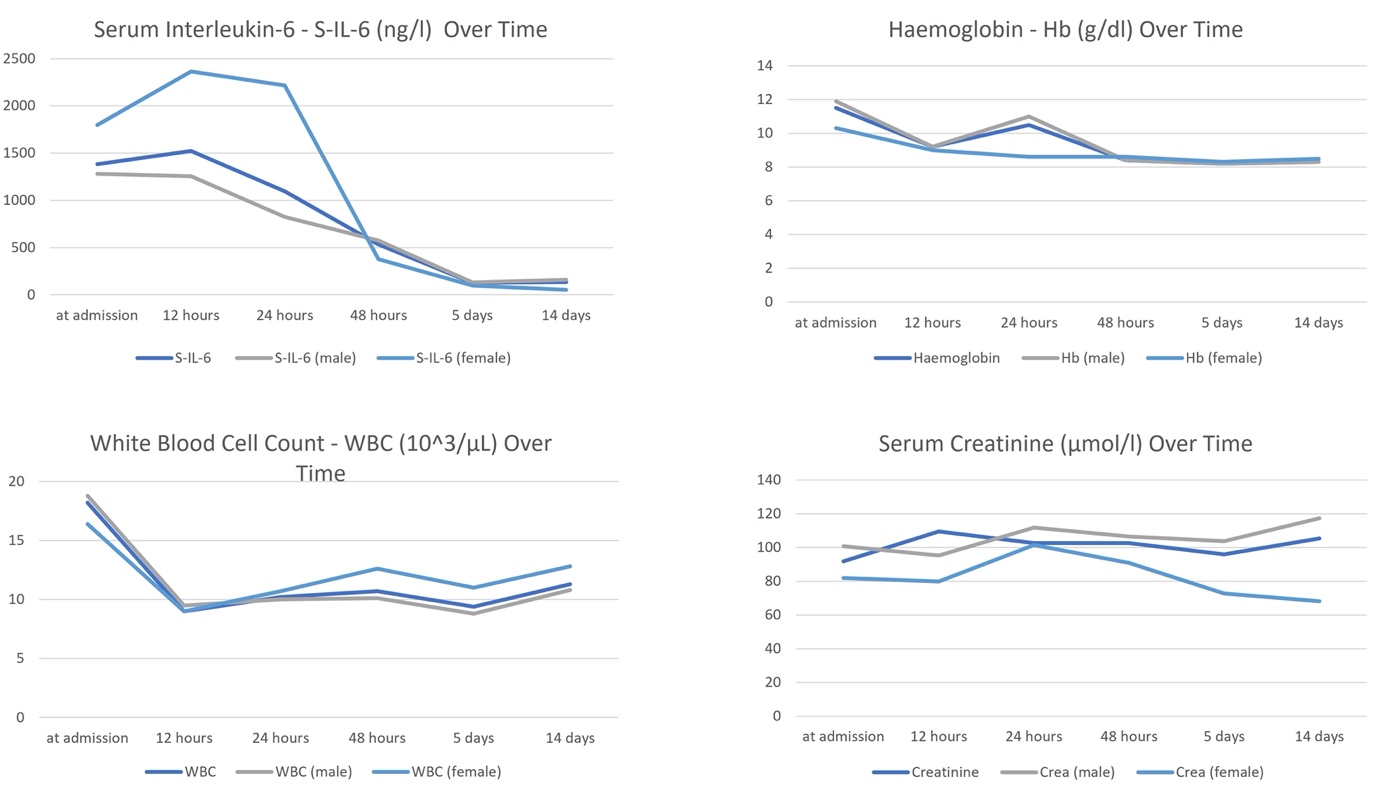


**Supplementary Data 1.** Sex Differences in Serum Creatinine Levels, White Blood Cell Count, Haemoglobin Levels and Interleucin 6 (IL-6) Levels over time. Time Point at admission, after 12 hours, after 24 hours, after 48 hours, after 5 days, after 14 days.


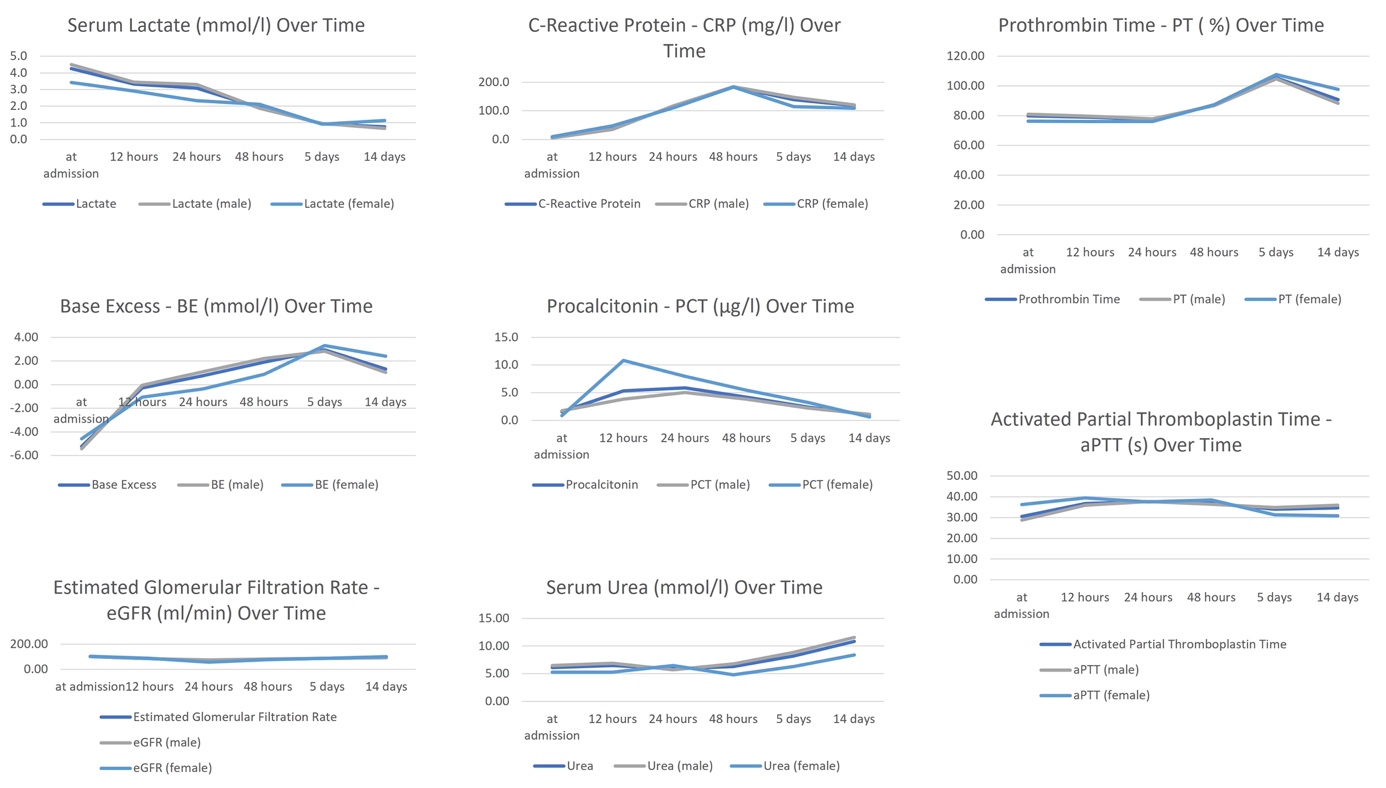


**Supplementary Data 2.** Sex Differences in Serum Lactate, Base Excess (BE), Estimated Glomerular Filtration Rate (eGFR), Serum Urea, Procalcitonin (PCT), C-Reactive Protein (CRP), Prothrombin Time (PT) and Activated Partial Thromboplastin Time (aPTT) over time. Time Point at admission, after 12 hours, after 24 hours, after 48 hours, after 5 days, after 14 days.


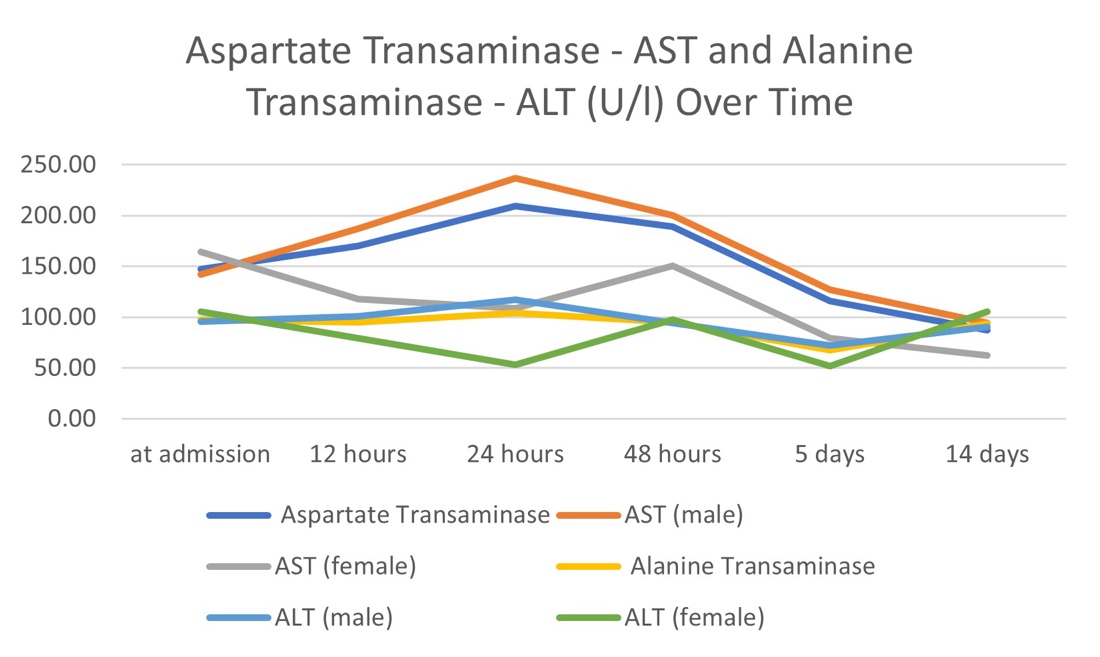


Supplementary Data 3. Sex Differences in aspartate transaminase (AST) and alanine transaminase (ALT) over time. Time Point at admission, after 12 hours, after 24 hours, after 48 hours, after 5 days, after 14 days.
